# Identifying cluster profiles based on barriers and facilitators to physical activity during COVID-19 confinement: A cross-sectional study using machine learning analysis

**DOI:** 10.1371/journal.pone.0354036

**Published:** 2026-07-27

**Authors:** Fernanda Castro Monteiro, Maria Luiza C. Wuillaume, Carlos Linhares Veloso Filho, Karla Figueiredo, Felipe Barreto Schuch, Andrea Nunes de Carvalho, Thiago Sousa Matias, Henrique Nunes Pereira Oliva, Renato Sobral Monteiro-Junior, Lara Carneiro, Andrea Camaz Deslandes

**Affiliations:** 1 Psychiatry Institute, Federal University of Rio de Janeiro, Rio de Janeiro, Brazil; 2 Institute of Health Sciences, Universidad de O’Higgins, Rancagua, Chile; 3 National Institute of Technology, Rio de Janeiro, Brazil; 4 Rio de Janeiro State University, Rio de Janeiro, Brazil; 5 Federal University of Santa Maria, Santa Maria, Brazil; 6 Faculty of Health Sciences, Universidad Autónoma de Chile, Providência, Chile; 7 Institute for Physical Activity and Nutrition, Deakin University, Geelong, Australia; 8 Institute for Mental and Physical Health and Clinical Translation (IMPACT), Deakin University, Geelong, Australia; 9 Federal University of Santa Catarina, Florianópolis, Brazil; 10 Department of Psychiatry, Yale University School of Medicine, New Haven, Connecticut, United States of America; 11 Physical Education Department, Montes Claros State University, Montes Claros, Minas Gerais, Brazil; 12 Physical Education Department, College of Education, United Arab Emirates University, Al Ain, Abu Dhabi, United Arab Emirates; Federal University of Pernambuco: Universidade Federal de Pernambuco, BRAZIL

## Abstract

Social restrictions, such as confinement periods, tend to reduce physical activity (PA) levels. However, sociodemographic factors may influence specific barriers and facilitators to PA during such periods. This study aimed to identify cluster profiles of individuals based on barriers and facilitators to physical activity (PA) during COVID-19 confinement. Brazilian adults participated in a cross-sectional online survey. The questionnaire collected demographic data, PA levels, sedentary behavior (SB), and perceived barriers and facilitators for PA. During data preprocessing, correlated barriers and facilitators related to a similar topic were aggregated. Using machine learning analysis, the K-modes evaluated by the Silhouette Score were used for barriers and the ROCK evaluated by the Silhouette Score was used for facilitators. The barriers model produced well-defined profiles, whereas the facilitators model did not. The facilitator model generated clusters with multiple negative silhouette coefficients and exhibited a significantly less cohesive cluster structure. Therefore, only the barriers-based model was used for further analysis. The best model generated eight clusters, each named according to the most frequent barriers in the group, such as “Inactive depressive women”, “Active depressive women” and “Super active”. The depressive clusters presented more barriers to PA, three barriers each one. Significant differences in PA and SB were observed across clusters. This work highlights the novelty of using unsupervised machine learning to uncover latent subgroups based on multiple concurrent barriers. In conclusion, tailored home-based and outdoor strategies should be developed, particularly targeting individuals with depressive symptoms and those facing significant time constraints.

## 1. Introduction

Critical periods of confinement, such as during climate changes, natural disasters, or pandemics, may profoundly disrupt daily life [1,2]. The most recent large-scale example was the COVID-19 pandemic. While the restrictive measures decreased viral circulation, they negatively impacted health behaviors like sleep, eating habits, physical activity (PA), and sedentary behavior (SB) [1,3]. Beyond these physical consequences, such situations frequently exacerbate mental health problems, including stress, fear, depression, and anxiety [3], which can further act as barriers to PA [2,4].

According to a cross-sectional study involving 1570 Brazilian participants during the pandemic, the most prevalent barriers to PA during this period were “lack of appropriate facilities/equipment/space” (17.4%), and “lack of time” (13.0%) [5]. Moreover, lack of motivation (OR=1.49; 95% CI = 1.19–1.86) and lack of appropriate facilities/equipment/space (OR=2.11; 95% CI = 1.57–2.83) were the factors most strongly associated with reduced PA levels [5]. In an online survey conducted among individuals in Canada and the US, Marashi et al. reported that many individuals were unmotivated to exercise due to anxiety (+8%), limited social support (+6%), or limited access to equipment (+23%) or space (+41%) during the social isolation [6]. Similarly, a Brazilian survey found the largest percentage in reported barriers was “Places to exercise are distant or inaccessible” (+ 37.5%), “Physical activity is hard work for me” (+ 18.5%), “I perceive barriers to physical activity practice” (+ 18.5%) [7]. Natural disasters can have similar effects: Li, Yang & An [8] documented decreased PA and increased alcohol consumption in Puerto Rico following a hurricane, highlighting the vulnerability of healthy lifestyle behaviors during such events.

In recent years, machine learning methods have gained prominence in health research, offering an alternative to traditional statistical approaches [9]. These methods are classified as supervised or unsupervised [10]. Unsupervised algorithms operate without labeled data, uncovering hidden patterns, structures, or clusters within datasets [11,12]. Among these, K-means clustering is one of the most widely used. It requires the number of clusters to be specified or estimated and groups data points based on similarity measures [11,12].

Applications of machine learning in PA research are increasing. For example, one observational study used K-means clustering to categorize individuals by PA levels measured with accelerometers. The more active cluster showed different types of PA on weekends than the less active cluster, and both clusters showed similar PA patterns during the week, differing only in intensity [13]. Another study compared machine learning methods with traditional cut-point approaches for predicting PA intensity in preschool-aged children, finding that machine learning models outperformed cut-point methods, particularly under free-living conditions [14]. Similarly, Nawrin et al. (2023) applied unsupervised clustering to step-count patterns (all-day, bi-phasic, morning, evening, irregular morning, and irregular night) that provided robust behavioral classifications [15].

Despite these advances, there seems to be a lack of studies using machine learning to investigate barriers to exercise, particularly during critical and challenging periods for some populations. Machine learning offers advantages over traditional statistical methods by identifying non-linear relationships and complex interactions among variables, which can lead to more nuanced and data-driven classifications of individuals. These profiles can be interpreted within existing theoretical frameworks, such as socio-ecological models, to better understand the multi-level influences on PA behavior.

The Socio-Ecological Model (SEM) provides a robust framework for understanding the complex interplay between individual, interpersonal, environmental, and policy-level factors that influence health behaviors. Evidence suggests that barriers to PA are not isolated to personal choices but are deeply embedded in a multi-level structure; for instance, intrapersonal factors like lack of motivation and depression symptoms often interact with interpersonal social support and environmental constraints, such as lack of access to safe facilities [16,17]. During periods of social restriction, these multi-level barriers become even more pronounced, as the home environment and community-level policies directly dictate the opportunities for movement [18]. By identifying clusters of individuals who share similar barrier profiles, this study aligns with the SEM’s premise that interventions must be tailored to address the specific combination of determinants – ranging from psychological states to environmental limitations – that characterize different population segments.

Understanding why specific groups remain less active is crucial for crafting tailored interventions. Factors such as socioeconomic status, access to safe exercise environments, and individual motivation can all play critical roles. In this context, the present study aimed to identify distinct individual profiles based on self-reported barriers to PA during COVID-19 social restriction. A secondary aim was to compare PA and sedentary behavior (SB) levels across these identified clusters.

## 2. Methods

This cross-sectional study was conducted via an online survey delivered using the Google Forms web survey platform (Google LLC, Mountain View, CA, United States). It is important to note that online recruitment through social media platforms introduces a selection bias towards digitally connected individuals, which may impact the generalizability of the findings [19,20].

Eligible participants were Brazilian residents aged between 18–85 years, of any gender, residing in any region of the country, and belonging to any social class. Brazilian individuals living outside Brazil and/or illiterate were excluded. Participants were recruited through online dissemination strategies, including social media platforms (E-mail, Facebook, WhatsApp, and Instagram) and invitations circulated via established researcher networks. Given the constraints imposed by the COVID-19 pandemic, particularly restrictions on in-person contact, we employed a snowball sampling approach, whereby initial participants were encouraged to share the study invitation within their own networks. This strategy was intentionally adopted to maximize reach and increase the number of respondents under conditions that limited the feasibility of probabilistic sampling. After providing electronic written informed consent, participants completed a multiple-choice questionnaire directly through the survey link (https://forms.gle/KDHKDpEopj2r1sxP8). Data collections were conducted between June and August 2020. Precisely, it started in 25/06/2020 and finished in 31/08/2020. The study protocol was approved by the Ethics Committee of the Psychiatry Institute, Federal University of Rio de Janeiro (register number 31739120.0.0000.5263).

### 2.1 Instruments

The questionnaire was applied once per participant and consisted of 40 questions covering demographic data, PA, sedentary behavior (SB), barriers, and facilitators for PA. The questions were about participant perceptions before and during the COVID-19 pandemic.

Self-reported diagnosis was obtained with the question: “Do you have any diagnosis of physical or mental illness?” In the present study, “Healthy” individuals were defined as the absence of any self-reported physical or mental illness diagnosis.

Sedentary behavior was assessed through one question before and one question during the pandemic: How many hours did the participant spend in sedentary time (zero to 2 hours; 2–4 hours; 4–6 hours; 6–8 hours; more than 8 hours). For analysis, responses were dichotomized as <8 hours/day or ≥8 hours/day, based on prior literature [21]. It is important to note that this single-item measure of sedentary behavior lacks formal validation, which is a limitation of the study.

Physical activity was assessed through four questions before and four questions during the pandemic: 1) Through a multiple-choice answer the participant indicated whether they practiced any type of activity and what this was. 2) Through a one-choice response the participant indicated the weekly frequency of activity (1–3 times; 3–5 times; 5–7 times). 3) Through a one-choice response, the participant indicated the intensity (light; moderate; vigorous). 4) Through a one-choice response, the participant indicated the duration (zero to 30 minutes; 30–60 minutes; 60–90 minutes; more than 90 minutes). We selected these options based on the World Health Organization (WHO) and American College of Sports Medicine (ACSM) recommendations, which advise adults to engage in at least 150 minutes/week of moderate-intensity or 75 minutes/week of vigorous-intensity activity, ideally in 20–60 minute sessions on most days [22].

Facilitators were assessed with two questions: one covering the period before COVID-19 (*What characteristics of physical activity would you consider facilitating your practice before COVID-19?*). The answers were: outdoor activities; activities with professional guidance; activities without professional guidance; group activities; activities with music; family activities; activities at home; space at home for physical activity practice and equipment at home for physical activity practice*.* The other question was about perception during the pandemic (*What characteristics of physical activity do you consider facilitating your practice today, during social isolation?*). The answers were: outdoor activities; activities with professional guidance; activities without professional guidance, virtually; group activities; activities with music; family activities; activities at home; space at home for physical activity practice; equipment at home for physical activity practice; equipment at home for physical activity practice.

Barriers were similarly assessed before and during the pandemic: (*What characteristic of physical activity would you consider as a barrier to your practice before COVID-19?*). The answers options were: *Physical activity took too much time from family responsibilities*; *Physical activity tired me; Places to exercise were far away/distant; It costs too much money to exercise; I had no convenient schedule for practicing physical activity; Physical activity was hard work for me; I had no interest in physical activity practice; I perceived barriers to physical activity practice*.

The other question was about perception during the pandemic (*What characteristic of physical activity do you consider as a barrier to your practice currently, during social isolation?*). The answers options were: *Physical activity takes too much time from family responsibilities; Physical activity tires me; Places to exercise are distant or inaccessible and posed a high risk at the time; It costs too much money to exercise; I have no convenient schedule for practicing physical activity; Physical activity is hard work for me; I have no interest in physical activity practice; I perceive barriers to physical activity practice*. All the questions presented a multiple-answer option.

To reduce dimensionality and simplify interpretation, conceptually similar barriers were aggregated. This decision was based on a qualitative review of the items and their semantic overlap. The barriers “physical activity tires me” and “physical activity is hard work for me” were combined into a new variable named “Physical activity is hard.” Similarly, “physical activity takes too much time from family responsibilities” and “I have no convenient schedule for physical activity” were aggregated into “Physical activity takes time.” The six barriers maintained in the dataset were: 1. *Physical activity is hard*; 2*. Physical activity takes time;* 3. *I perceive barriers to physical activity practice;* 4. *I have no interest in practicing physical activity;* 5. *Places to exercise are inaccessible and/or closed and posed a high risk at the time*; 6. *It costs too much money to exercise.*

The answers were affirmative sentences based on the *Exercise Barriers/Benefits Scale (EBBS)*, a Likert-type validated scale [23] (Further details are included in Monteiro et al., 2023) [7]. The EBBS is a scale that assesses the participant’s perception of barriers and benefits for exercise (see S1 File). However, in our study, the answers were modified for the COVID-19 pandemic scenario and social isolation.

### 2.2 Data processing

A total of 1252 responses were received. Six were excluded due to missing data and 32 due to contradictory PA responses during the pandemic, leaving 1214 participants for analysis. “Contradictory responses” referred to participants who reported engaging in zero minutes of physical activity but simultaneously selected a high weekly frequency and intensity, or vice versa. Barriers and facilitators to PA (binary and categorical variables) were grouped based on similarities, forming aggregated sets. The preprocessing also included consolidating identical records, resulting in 33 distinct combinations of barriers and 50 of facilitators. Following the clustering step, all 1214 records were assigned to their respective groups. Due to the confinement period characteristics and nature, we did not perform sample size calculation for this study. However, although no priori sample size calculation was performed, we conducted a post hoc power analysis using the G*Power software (one-way ANOVA, 8 groups, α = 0.05). With the obtained sample size (N = 1,214), the study has 71.3% power to detect a small effect (f = 0.10) and 100% power to detect a medium effect (f = 0.25) across clusters. This level of power is considered adequate for exploratory studies, especially those employing machine learning techniques on samples collected under natural conditions (pandemic). Therefore, the sample size is sufficient to support the analyses performed and the conclusions presented.

Clustering was performed using the K-modes and ROCK algorithms, combined with the adapted Silhouette Score and the Calinski-Harabasz index. Dissimilarity between two records was defined as the number of variables in which their categorical values differed; this dissimilarity matrix was precomputed and used as input to both clustering algorithms and to the adapted evaluation metrics. All analyses were conducted in R (version 4.1.1), using the package proxyC (version v0.1.0). To assess the stability of the clustering solutions, we adopted a multi-run strategy combined with internal validation metrics. Since clustering is an unsupervised task, traditional cross-validation procedures are not applicable; therefore, solution robustness was evaluated based on consistency across repeated runs and parameter configurations.

For the K-modes algorithm, 1,000 runs were performed for each value of k (tested across the full range of 2–33 clusters for the barriers dataset and 2–50 clusters for the facilitators dataset) using different random initializations (seeds). This procedure was implemented to reduce sensitivity to initial conditions, as the algorithm may converge to different local optima. For each k, the final solution was selected based on the average performance across internal validation metrics, and the overall best k for each dataset corresponded to the value that maximized this average — k = 8 for the barriers dataset, identified as the global maximum of the adjusted Silhouette Score curve (Silhouette Score = 0.4446).

For the ROCK algorithm, stability was evaluated through a systematic exploration of its structural parameters. Specifically, combinations of the number of clusters (k), neighborhood threshold (θ), and similarity functions were tested. Fifteen candidate θ values, ranging from 0 to 0.99, were evaluated in combination with five similarity functions (cosine, Jaccard, Hamman, simple matching, and faith similarity), with dissimilarity computed as one minus each similarity value. The parameter configuration that maximized the internal validation metrics was selected (θ = with the cosine similarity function, yielding an adjusted Silhouette Score of 0.2714/ (θ = with the simple matching similarity function, yielding adjusted Calinski-Harabasz index of 1.065), and subsequent analyses were conducted by varying k under these optimized conditions.

To aid in identifying profiles, relative frequency histograms were generated using variables from the datasets that were not utilized to train the models. It is important to highlight that when barriers were utilized for clustering, the aggregated facilitators were employed for profiling, and conversely, when clustering was based on facilitators, aggregated barriers were incorporated into the profiling analysis. To decide on the best clustering, not only was the performance evaluated by the chosen metric assessed, but also on interpretability and practical suitability of the findings.

After a thorough comparison of clusters using the given plots, we arrived at a significant conclusion. The barriers model proved to be more effective in creating well-defined target profiles, unlike the facilitators model. The details are described below:

#### 2.2.1 Detailed rationale for excluding the facilitator model.

The decision to exclude the facilitator model from the study’s main analysis was based on a rigorous assessment of the quality of the clusters and the interpretability of the results. Although a parallel analysis was attempted using the ROCK algorithm for the facilitator data, the results did not meet the criteria for robustness and interpretability necessary to proceed with the analysis.

***Numerical and qualitative evidence:*** Barrier Model (K-modes): Analysis of the Adjusted Silhouette Coefficient for the barrier model demonstrated good clustering quality. All records exhibited positive silhouette coefficients, indicating that each individual was well-assigned to their cluster and well-separated from neighboring clusters. The best silhouette score was 0.4432984, confirming the good cohesion and separation of the 8 identified clusters.

Facilitator Model (ROCK): In contrast, the facilitator model exhibited a significantly less cohesive cluster structure. A notable presence of bars crossing the zero line into the negative side was observed. Negative silhouette coefficients indicate that individuals are closer to a neighboring cluster than to their own cluster, suggesting misallocation or that the individual may be an outlier. The high incidence of negative coefficients and the absence of a clear clustering structure indicated that the ROCK algorithm failed to form cohesive and well-separated clusters for the facilitator data. There were 11 negative values out of 50, meaning that 22% of the data points were misclassified. This means that about 22% of the people (11 out of 50) fall into this category. Besides, the best reported Silhouette value for the ROCK scenario is 0.2762202.

In addition to the quantitative metrics, the qualitative expert assessment revealed that the facilitators model did not produce meaningful or interpretable profiles. The facilitators patterns did not cluster in a way that could be translated into distinct and clinically relevant profiles. This contrasts sharply with the barrier clusters, which exhibited clear characteristics useful for developing targeted interventions.

In light of this, we focused our further analysis only on the model created with the barriers. All analyses were conducted in R, with a dissimilarity matrix employed to facilitate the clustering process.

## 3. Results

A total of 1214 individuals were included in this study. Most of the sample was composed of females (73.3%), aged between 36–44 years old (26.2%), married (51.1%) and with post-graduation completed (51%). Among self-reported diagnoses, anxiety disorder (9.3%) and depression (3.2%) were the most prevalent (Table 1). Most of the sample was physically active (73.7%) and regarding SB, most of the sample presented less than 8 hours/day of sitting time (64.7%) (Table 1).

**Table 1:** Demographic characteristics.

	Total sample (N = 1214)
Variable	N (%)
**Sex**	
Female	889 (73.3%)
Male	352 (26.7%)
**Age**	
18 to 26 years old	205 (17%)
27 to 35 years old	253 (20.8%)
36 to 44 years old	318 (26.2%)
45 to 53 years old	213 (17.5%)
54 to 62 years old	145 (12%)
63 to 71 years old	68 (5.6%)
72 to 80 years old	11 (0.9%)
**Income range**	
No income	5 (0.4%)
Until 1 minimum wages	22 (1.8%)
1-3 minimum wages	182 (15%)
3-6 minimum wages	280 (23%)
6-9 minimum wages	240 (19.8%)
More than 9 minimum wages	485 (40%)
**Marital status**	
Single	460 (38%)
Married	621 (51.1%)
Divorced	111 (9.1%)
Widowed	22 (1.8%)
**Scholarity**	
Complete college	48 (4%)
Incomplete college	546 (45%)
Postgraduate	620 (51%)
**Diagnosis**	
Healthy	1063 (87.5%)
Anxiety	113 (9.3%)
Depression	38 (3.2%)
**Physical activity practice**	
No	320 (26.3%)
Yes	894 (73.7%)
**Sedentary behavior**	
<8h sitting	786 (64.7%)
>= 8h sitting	428 (35.3%)

**Fig 1:**
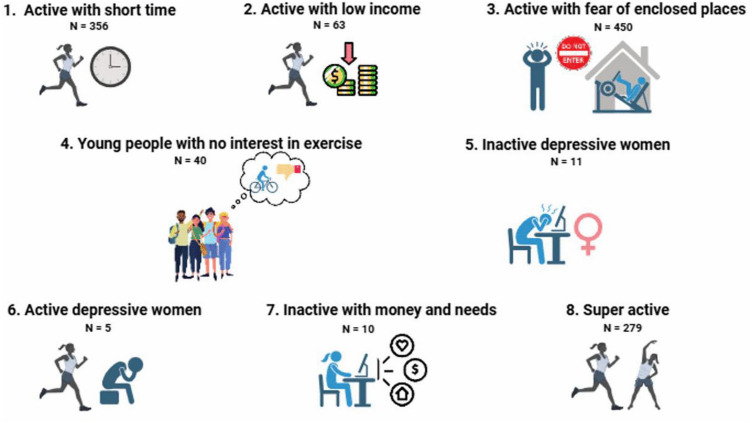
Most frequent barriers for each cluster – barriers clustering.

Based on the self-reported barriers to PA during the pandemic, profiles of clusters of individuals were created. The number of clusters evaluated ranged from 2 to 33 for the barriers dataset and the optimal number of clusters was k = 8, which represented the global maximum of the adjusted Silhouette Score curve (Silhouette Score = 0.4446). This means that the best barrier clustering model generated 8 clusters whose profiles were named according to the variables in which they had unusual distributions and according to the most frequent barriers in the group. For example, clusters composed by high prevalence of physical activity practice were named as “active” plus the most frequent barriers in the group (clusters 1, 2,3). Those composed only by females with self-reported depression diagnosis were named accordingly (clusters 5 and 6). The cluster composed by young people’s high prevalence and the most frequent barrier reported was “lack of interest in exercise” were named accordingly (cluster 4). The group with a high prevalence of physically inactive individuals and a high prevalence of high-income reported barriers related to the time available for exercise and the financial resources required to do so (cluster 7). The more active group did not report any barriers to exercise and was named accordingly (cluster 8). The profiles generated were: 1) Active with short time; 2) Active with low income; 3) Active with fear of enclosed places; 4) Young people with no interest in exercise; 5) Inactive depressive women; 6) Active depressive women; 7) Inactive with money and needs; 8) Super active (Fig 1 and Table 2).

**Table 2:** Clusters demographics characteristics.

	1. Active with short time	2. Active with low income	3. Active with fear of enclosed places	4. Young people with no interest in exercise	5. Inactive depressive women	6. Active depressive women	7. Inactive with money and needs	8. Super active
**N (%)**	356 (29.3%)	63 (5.1%)	450 (37%)	40 (3.5%)	11 (0.9%)	5 (0.5%)	10 (0.8%)	279 (23%)
**Sex – N (%)**								
Female	278 (78.1%)	50 (79.3%)	315 (70%)	35 (87.5%)	11 (100%)	5 (100%)	5 (50%)	190 (68.1%)
Male	78 (21.9%)	13 (20.7%)	135 (30%)	5 (12.5%)			5 (50%)	89 (31.9%)
**Age – N (%)**								
18 to 26 years old	36 (10.1%)	6 (9.5%)	58 (12.9%)	8 (20%)	4 (36.4%)	–	–	93 (33.3%)
27 to 35 years old	70 (19.7%)	17 (27%)	96 (21.3%)	7 (17.5%)	3 (27.3%)	–	4 (40%)	56 (20.1%)
36 to 44 years old	118 (33.1%)	15 (23.8%)	130 (28.9%)	7 (17.5%)	1 (9.1%)	2 (40%)	1 (10%)	44 (15.8%)
45 to 53 years old	71 (19.9%)	10 (15.9%)	74 (16.4%)	6 (15%)	2 (18.2%)	–	3 (30%)	47 (16.8%)
54 to 62 years old	38 (10.7%)	12 (19%)	49 (10.9%)	12 (30%)	1 (9.1%)	1 (20%)	2 (20%)	30 (10.8%)
63 to 71 years old	20 (5.6%)	2 (3.2%)	37 (8.2%)	–	–	2 (40%)	–	7 (2.5%)
72 to 80 years old	3 (0.8%)	1 (1.6%)	5 (1.1%)	–	–	–	–	2 (0.7%)
More than 80 years old	–	–	1 (0.2%)	–	–	–	–	–
**Income range – N (%)**								
No income	2 (0.6%)	–	1 (0.2%)	–	–	1 (20%)	–	1 (0.4%)
Until 1 minimum wages	4 (1.1%)	4 (6.3%)	9 (2%)	–	–	–	–	5 (1.8%)
1-3 minimum wages	41 (11.5%)	9 (14.3%)	62 (13.8%)	5 (12.5%)	2 (18.2%)	–	–	63 (22.6%)
3-6 minimum wages	79 (22.2%)	15 (23.8%)	87 (19.3%)	7 (17.5%)	4 (36.4%)	1 (20%)	5 (50%)	82 (29.4%)
6-9 minimum wages	67 (18.8%)	9 (14.3%)	81 (18%)	11 (27.5%)	2 (18.2%)	1 (20%)	3 (30%)	66 (23.7%)
More than 9 minimum wages	163 (45.8%)	26 (41.3%)	210 (46.7%)	17 (42.5%)	3 (27.3%)	2 (40%)	2 (20%)	62 (22.2%)
**Marital status**								
Single	118 (33.1%)	21 (33.3%)	155 (34.4%)	15 (37.5%)	7 (63.6%)	–	–	144 (51.6%)
Married	201 (56.5%)	38 (60.3%)	242 (53.8%)	19 (47.5%)	2 (18.2%)	5 (100%)	6 (60%)	108 (38.7%)
Divorced	29 (8.1%)	2 (3.2%)	45 (10%)	6 (15%)	2 (18.2%)	–	6 (40%)	23 (8.2%)
Widowed	8 (2.2%)	2 (3.2%)	8 (1.8%)	–	–	–	–	4 (1.4%)
**Scholarity**								
Incomplete college	7 (2%)	3 (4.8%)	18 (4%)	1 (2.5%)	2 (18.2%)	–	–	17 (6.1%)
Complete college	153 (43%)	19 (30.2%)	184 (40.9%)	16 (40%)	6 (54.5%)	1 (20%)	3 (30%)	164 (58.8%)
Postgraduate	196 (55.1%)	41 (65.1%)	248 (55.1%)	23 (57.5%)	3 (27.3%)	4 (80%)	7 (70%)	98 (35.1%)
**Self-reported depression diagnosis**	10 (2.8%)	3 (4.8%)	17 (3.8%)	2 (5%)	11 (100%)	5 (100%)	–	6 (2.2%)

The largest clusters corresponding to 89.3% of the sample were “Active with short time”, “Active with fear of enclosed places” and “Super active”. The most prevalent barriers were “Physical activity takes time”, “I have no interest in practicing physical activity” and “Places to exercise as inaccessible and/or closed and posed a high risk at the time”. Clusters with more prevalence of SB and PI showed more barriers, while the most active cluster did not report any type of barriers to PA. The cluster with fear of enclosed places did not show high levels of SB and physical inactivity (PI). There were two clusters with a higher prevalence of self-reported depressive diagnosis, both with more barriers to PA than the other clusters. Although both groups reported “I have no convenient schedule for physical activity/Physical activity takes time” and “I have no interest in practicing physical activity”, the clusters differ in one barrier perceived. While a more inactive depressed cluster informed that “Physical activity is hard”, the more active cluster considers “Places to exercise as inaccessible and/or closed and posed a high risk at the time” a barrier.

Regarding clusters sociodemographic characteristics, the S1 Table presents all pairwise comparisons between the eight clusters for each variable, with p-values adjusted using the Bonferroni correction for 28 comparisons (α = 0.05/28 = 0.00178) (full detail in supplemental material). After correction, significant differences were observed primarily for comparisons involving Cluster 8 (super active), which differed significantly from Clusters 1, 2, 3, and 7 for age, marital status, income, and education (p < 0.00178). Cluster 6 (active depressive women) also showed significant differences from multiple clusters for age and income. Cluster 5 (inactive depressive women) differed significantly from Cluster 1 for education (p = 0.001). No significant differences were found between Clusters 2 and 3 for any variable, nor between Clusters 4 and 8 for most variables. A small number of comparisons (e.g., Cluster 5 vs. Cluster 6 for sex and marital status, Cluster 2 vs. Cluster 5 for education) reached nominal significance (p < 0.05) but did not maintain after Bonferroni correction.

Considering the clusters profiles, significant differences were found in SB levels between the groups. The “Super active” group was significantly less sedentary than the following groups: “Active with short time” group (p ≤ 0.001); “Active with low-income” (p ≤ 0.001); “Active with fear of enclosed places” (p ≤ 0.001); “Young people with no interest in exercise” (p ≤ 0.001) and the “Inactive with money and needs” group (p ≤ 0.001). Regarding PA levels, the same was observed. The “Super active” group was significantly more active than six groups: “Active with short time” group (p ≤ 0.001); “Active with low-income” (p ≤ 0.001); “Active with fear of enclosed places” (p ≤ 0.001); “Young people with no interest in exercise” (p ≤ 0.001); “Inactive depressive women” (p ≤ 0.001) and the “Inactive with money and needs” group (p ≤ 0.001).

Moreover, the largest clusters were the most active and the least sedentary: “Active with short time”, “Active with fear of enclosed places” and “Super active” (Tables 3 and 4). Regarding the active clusters, the depressive women group was the one that presented the lower prevalence of self-reported PA, while “Active with lower income” was the group that spent more time in SB (Tables 3 and 4). Regarding the inactive groups, the “Inactive with money and needs” group was the most sedentary (Table 3).

**Table 3:** Sedentary behavior among clusters profiles.

	Sedentary Behavior (sitting more than 8 hours per day) Yes/No	Active with short time	Active with low-income	Active with fear of enclosed places	Young people with no interest in exercise	Inactive depressive women	Active depressive women	Inactive with money and needs	Super actives
**Clusters profiles**									
1. Active with short time (N = 356)	39% − 61%	–	X² = 1.088; df = 1; p = 0.297	X² = 0.135; df = 1; p = 0.713	X² = 1.072; df = 1; p = 0.300	(-) p = 0.354	(-) p = 1.000	(-) p = 0.096	X² = 6.462; df = 1; p ≤ 0.001**
2. Active with low-income (N = 63)	46% − 54%	–	–	X² = 1.586; df = 1; p = 0.056	X² = 0.021; df = 1; p = 0.884	(-) p = 0.747	(-) p = 1.000	(-) p = 0.190	X² = 18.545; df = 1; p ≤ 0.001**
3. Active with fear of enclosed places (N = 450)	38% − 62%	–	–	–	X² = 1.465; df = 1; p = 0.226	(-) p = 0.347	(-) p = 1.000	(-) p = 0.050*	X² = 25.241; df = 1; p ≤ 0.001**
4. Young people with no interest in exercise (N = 40)	48% − 52%	–	–	–	–	(-) p = 0.743	(-) p = 1.000	(-) p = 0.294	X² = 14.635; df = 1; p ≤ 0.001**
5. Inactive depressive women (N = 11)	55% − 45%	–	–	–	–	–	(-) p = 1.000	(-) p = 0.659	(-) p = 0.014*
6. Active depressive women (N = 5)	40% − 60%	–	–	–	–	–	–	(-) p = 0.329	(-) p = 0.271
7. Inactive with money and needs (N = 10)	70% − 30%	–	–	–	–	–	–	–	(-) p ≤ 0.001**
8. Super actives (N = 279)	20% − 80%	–	–	–	–	–	–	–	–

Note: X² = Qui-squared test; df = degrees of freedom; p = p value; (-) = Fisher’s test.

** p < 0.00178 (significant after Bonferroni correction for 28 comparisons).

* p < 0.05 (significant before correction, but not after Bonferroni).

**Table 4:** Physical activity practice among clusters profiles.

	Physical Activity (practice) Yes/No	Active with short time	Active with low-income	Active with fear of enclosed places	Young people with no interest in exercise	Inactive depressive women	Active depressive women	Inactive with money and needs	Super actives
**Clusters profiles**									
1. Active with short time (N = 356)	65% − 35%	–	X² = 0.486; df = 1; p = 0.486	X² = 12.457; df = 1; p ≤ 0.001**	X² = 6.093; df = 1; p = 0.014*	**(-)** p ≤ 0.003**	(-) p = 1.000	(-) p = 0.177	X² = 59.292; df = 1; p ≤ 0.001**
2. Active with low-income (N = 63)	62% − 38%	–	–	X² = 7.315; df = 1; p = 0.007*	X² = 2.314; df = 1; p = 0.128	(-) p = 0.019*	(-) p = 1.000	(-) p = 0.307	X² = 38.861 df = 1; p ≤ 0.001**
3. Active with fear of enclosed places (N = 450)	76% − 24%	–	–	–	X² = 18.463; df = 1; p ≤ 0.001**	**(-)** p ≤ 0.001**	(-) p = 0.598	(-) p = 0.017*	X² = 25.449; df = 1; p ≤ 0.001**
4. Young people with no interest in exercise (N = 40)	40% − 60%	–	–	–	–	(-) p = 0.166	(-) p = 0.652	(-) p = 1.000	X² = 59.027; df = 1; p ≤ 0.001**
5. Inactive depressive women (N = 11)	18% − 82%	–	–	–	–	–	(-) p = 0.245	(-) p = 0.361	**(-)** p ≤ 0.001**
6. Active depressive women (N = 5)	60% − 40%	–	–	–	–	–	–	(-) p = 0.608	(-) p = 0.073
7. Inactive with money and needs (N = 10)	40% − 60%	–	–	–	–	–	–	–	**(-)** p ≤ 0.001**
8. Super actives (N = 279)	91.3% − 8.7%	–	–	–	–	–	–	–	–

Note: X² = Qui-squared test; df = degrees of freedom; p = p value; (-) = Fisher’s test.

** p < 0.00178 (significant after Bonferroni correction for 28 comparisons).

* p < 0.05 (significant before correction, but not after Bonferroni).

## 4. Discussion

To the best of our knowledge, this is the first study to identify individuals´ cluster profiles according to barriers to PA among Brazilians during a worldwide critical period. Considering the cluster profiles, significant differences were found in SB and PA levels between the groups. The more representative cluster was active individuals who do not perceive barriers to PA, being the most active group *(“Super Actives”*). Considering the other clusters with the highest prevalence of PA (“*Active with short time”* and *“Active with fear of enclosed places”*), barriers such as lack of time and safety concerns were found, in line with other studies reporting on challenging situations such as health emergencies or economic crises [24]. In clusters of women with self-reported depression diagnosis, both groups presented the highest prevalence of barriers to PA (3 barriers each). The depressed active group reported barriers of lack of time, disinterest in exercise and fear of enclosed places, whereas, the inactive depressive women reported lack of time, disinterest in PA and PA is hard, presenting the highest prevalence of PI.

In accordance with our findings, disinterest in exercise could be an important barrier in people with mental illness, related to psychological aspects such as self-confidence and lower self-efficacy [25]. The bidirectional relationship between depression and physical inactivity is well-established in the literature, reflecting a complex and mutually reinforcing cycle [26–29]. On one hand, depressive symptoms, particularly anhedonia, fatigue, and psychomotor retardation, can substantially reduce motivation, perceived self-efficacy, and energy levels, making engagement in PA seem effortful and less rewarding [25,30,31]. These symptoms are also associated with alterations in reward processing and executive functioning, which further impair the initiation and maintenance of health behaviors such as regular PA [32]. On the other hand, physical inactivity contributes to the onset and worsening of depressive symptoms through multiple pathways, including dysregulation of neurobiological systems (e.g., reduced neurogenesis, impaired monoaminergic signaling, and increased inflammation) [33,34].

Indeed, higher depressive symptoms were more likely to endorse “lack of self-motivation” as a barrier to exercise in times of prolonged restriction or uncertainty [6,35]. These findings align with broader patterns observed in the general population, where women, in comparison to men, tend to encounter more impediments to exercise initiation and exhibit lower levels of PA [36]. Moreover, the restrictive measures implemented during crisis could exacerbate well-known health issues and social inequities within the female population, contributing to more perceived barriers to PA. Unlike our study, barriers such as poor mental and physical health, and low energy were also reported in previous literature [37] and may influence the decision to practice PA in this specific population.

Furthermore, the fear of exposure to health threats was also found in other studies as a barrier to PA [7,24]. However, in our study, it seems that this barrier did not influence PA practice. More broadly, the role of the environment as a barrier or facilitator can significantly influence individuals’ likelihood of engaging in physical activity, especially during times when normal routines are disrupted [2,8,20]. Other key predictors of sustained PA during lockdown could be explained by factors such as higher education, higher income and living in urban areas, reinforcing existing knowledge about the demographic and socio-economic factors that could influence exercise habits [38] and could support our main findings. On the other hand, the cluster *“Inactive with money and needs*” was the most sedentary, ranking as the second least active and contradicting this finding, despite its high-income characteristics.

Meta-analyses have confirmed a well-known pattern of decreased physical activity levels during periods of widespread disruption, like the COVID-19 pandemic, across various ages and populations globally, highlighting the difficulties of maintaining active behavior during such times [19,20]. In fact, limited access to public spaces and exercise opportunities may foster SB levels. Although it was not the main goal of our study, the literature indicates that those who experienced PA habits before a crisis could present more self-efficacy and intrinsic motivation to deal with changes in daily exercise routine and maintain their practice during the pandemic [39].

The use of existing equipment and infrastructure to exercise were found as important facilitators to PA in restrictive contexts [7], but was not considered in the present study. Due to similarities in reported facilitators for PA in our sample, it was not possible to define and target profiles of specific individuals considering them. In other study in times of crisis, facilitators involving individual factors, environmental factors, and social domains were found, in line with our primary results [40].

Although machine learning models can potentially be an interesting tool for solving problems related to research in PA and SB, little has been explored in this field concerning crisis situations. A recent study during a large-scale restriction period aimed to evaluate PA levels during the lockdown and identify the factors influencing these levels [41]. A multivariate analysis was conducted to identify associations between different sociodemographic factors (such as age, sex, education level) and PA levels [41]. The analysis showed that factors such as outdoor activity and working on-site were associated with higher PA levels [41]. [41]

Similarly, Caldirola et al. (2022) [42] employed machine learning to analyze data collected during a major societal disruption of Italian adults with no preexisting psychiatric conditions. The model was based on several significant variables (low resilience, crisis-related stress, being an undergraduate student) that influenced its predictive capability. The findings revealed that a significant percentage of the participants developed new psychiatric disorders during the pandemic. The predictive model developed in the study has a moderate performance and showed a sensitivity of 70% and specificity of 73% [42].

Across systematic reviews conducted during the COVID-19 pandemic, several consistent barriers to PA have been identified. In accordance with our findings, environmental restrictions – such as lockdown measures, closure of recreational facilities, and reduced access to outdoor spaces – emerge as the most prominent structural constraints [43,44]. In the same way, psychosocial barriers, including reduced motivation, increased psychological distress, and lack of social support, further hinder engagement in PA [45]. Additionally, lifestyle disruptions – such as increased screen time, remote work demands, and loss of daily routines – contribute to more sedentary behaviors and reduced opportunities for movement [3,44].

Although current literature presents important advances and findings using machine learning, few studies address this methodology in relation to the practice of PA, specifically, using data from critical periods such as the COVID-19 pandemic. In light of this, our study holds particular relevance in informing that PA behavior may be linked to barriers to PA, and even active individuals could perceive this interaction. Our cluster analysis further refines this understanding by demonstrating how these barriers co-occur in specific subgroups, providing a more granular view for public health planning.

However, the present study has certain limitations. First, the lack of formal validation of the questionnaire used in the study. Second, the reliance on self-reported data introduces the potential for recall bias and subjectivity. Participants may interpret and respond to questions differently, impacting the accuracy of the results. Third, the nature of the study precludes more causal conclusions and may compromise the interpretability of the findings. Fourth, another important limitation of the study is the small sample size in some clusters, especially those composed of depressed individuals. Fifth, the use of a single question to assess self-reported diagnosis of mental illness limits the robustness of the findings. Furthermore, the sample presents a bias, due to its characteristics, as it does not represent the population expectation of PA in Brazil, which leads the reader to suggest that data on barriers to PA may also be biased and not represent the population.

## 5. Conclusion

In conclusion, the current study provides useful information on factors influencing PA. By identifying distinct clusters with specific barriers, it is possible to promote home-based exercise, improve access to outdoor recreational spaces while ensuring safety measures, and provide adequate support to these populations. Understanding the diverse challenges individuals face allows for more effective, tailored strategies to encourage exercise, considering unique subgroup needs. Overall, the findings contribute to evidence-based interventions to enhance PA and well-being during crises and other challenging circumstances.

## Supporting information

S1 File Exercise Barriers/Benefits Scale (EBBS). (PDF)

S1 Table All pairwise comparisons between clusters for all sociodemographic variables. (DOCX)
