# Supplementary material for: Identifying cluster profiles based on barriers and facilitators to physical activity during COVID-19 confinement: A cross-sectional study using machine learning analysis
Source: PLoS One. 2026 Jul 27;21(7):e0354036. doi: 10.1371/journal.pone.0354036 (PMC13405084; doi:10.1371/journal.pone.0354036)
Supplement: S1 File — (PDF) [file pone.0354036.s001.pdf]

EXERCISE BENEFITS/BARRIERS SCALE

DIRECTIONS: Below are statements that relate to ideas about exercise. Please indicate the degree to which you agree or disagree with the statements by circling SA for strongly agree, A for agree, D for disagree, or SD for strongly disagree.

		Strongly Agree	Agree	Disagree	Strongly Disagree
1.	I enjoy exercise.	SA	A	D	SD
2.	Exercise decreases feelings of stress and tension for me.	SA	A	D	SD
3.	Exercise improves my mental health.	SA	A	D	SD
4.	Exercising takes too much of my time.	SA	A	D	SD
5.	I will prevent heart attacks by exercising.	SA	A	D	SD
6.	Exercise tires me.	SA	A	D	SD
7.	Exercise increases my muscle strength.	SA	A	D	SD
8.	Exercise gives me a sense of personal accomplishment.	SA	A	D	SD
9.	Places for me to exercise are too far away.	SA	A	D	SD
10.	Exercising makes me feel relaxed.	SA	A	D	SD
11.	Exercising lets me have contact with friends and persons I enjoy.	SA	A	D	SD
12.	I am too embarrassed to exercise.	SA	A	D	SD
13.	Exercising will keep me from having high blood pressure.	SA	A	D	SD
14.	It costs too much to exercise.	SA	A	D	SD
15.	Exercising increases my level of physical fitness.	SA	A	D	SD
16.	Exercise facilities do not have convenient schedules for me.	SA	A	D	SD
17.	My muscle tone is improved with exercise.	SA	A	D	SD
18.	Exercising improves functioning of my cardiovascular system.	SA	A	D	SD
19.	I am fatigued by exercise.	SA	A	D	SD
20.	I have improved feelings of well being from exercise.	SA	A	D	SD
21.	My spouse (or significant other) does not encourage exercising.	SA	A	D	SD

(Continued on reverse side)

		Strongly Agree	Agree	Disagree	Strongly Disagree
22.	Exercise increases my stamina.	SA	A	D	SD
23.	Exercise improves my flexibility.	SA	A	D	SD
24.	Exercise takes too much time from family relationships.	SA	A	D	SD
25.	My disposition is improved with exercise.	SA	A	D	SD
26.	Exercising helps me sleep better at night.	SA	A	D	SD
27.	I will live longer if I exercise.	SA	A	D	SD
28.	I think people in exercise clothes look funny.	SA	A	D	SD
29.	Exercise helps me decrease fatigue.	SA	A	D	SD
30.	Exercising is a good way for me to meet new people.	SA	A	D	SD
31.	My physical endurance is improved by exercising.	SA	A	D	SD
32.	Exercising improves my self-concept.	SA	A	D	SD
33.	My family members do not encourage me to exercise.	SA	A	D	SD
34.	Exercising increases my mental alertness.	SA	A	D	SD
35.	Exercise allows me to carry out normal activities without becoming tired.	SA	A	D	SD
36.	Exercise improves the quality of my work.	SA	A	D	SD
37.	Exercise takes too much time from my family responsibilities.	SA	A	D	SD
38.	Exercise is good entertainment for me.	SA	A	D	SD
39.	Exercising increases my acceptance by others.	SA	A	D	SD
40.	Exercise is hard work for me.	SA	A	D	SD
41.	Exercise improves overall body functioning for me.	SA	A	D	SD
42.	There are too few places for me to exercise.	SA	A	D	SD
43.	Exercise improves the way my body looks.	SA	A	D	SD

© K. Sechrist, S. Walker, N. Pender, 1985. Reproduction without authors' express written consent is not permitted. Permission is obtainable by downloading the Exercise Benefits/Barriers Scale (EBBS) Information and Permission Letter from deepblue.lib.umich.edu. If additional information is needed, contact Dr. Karen Sechrist by e-mail: krsech@pacbell.net.

EXERCISE BENEFITS/BARRIERS SCALE

Scoring Information

The instrument may be scored and used in its entirety or as two separate scales. The instrument has a four-response, forced-choice Likert-type format with responses ranging from 4 (strongly agree) to 1 (strongly disagree). Barrier Scale items are reverse-scored. Items on the Barrier Scale are numbers 4, 6, 9, 12, 14, 16, 19, 21, 24, 28, 33, 37, 40 and 42.

Missing data may be handled in one of two ways. If more than five percent of the items are unanswered, it is recommended that the response be discarded. If the missing item response rate is less than five percent, median substitution prevents falsely low scores.

Scores on the total instrument can range from 43 to 172. The higher the score, the more positively the individual perceives exercise. When the Benefits Scale is used alone, the score range is between 29 and 116. When the Barriers Scale is used alone, scores range between 14 and 56. If used alone, the Barriers Scale does not need to be reverse-scored. In this instance, the higher the score on the Barriers Scale, the greater the perception of barriers to exercise.
