# Supplementary material for: Identifying cluster profiles based on barriers and facilitators to physical activity during COVID-19 confinement: A cross-sectional study using machine learning analysis
Source: PLoS One. 2026 Jul 27;21(7):e0354036. doi: 10.1371/journal.pone.0354036 (PMC13405084; doi:10.1371/journal.pone.0354036)
Supplement: S1 Table — (DOCX) [file pone.0354036.s002.docx]

**Table S1. All pairwise comparisons between clusters for all sociodemographic variables (Bonferroni-corrected p-values).**

| **Pair** | **Sex** | **Age** | **Marital status** | **Income range** | **Scholarity** |
| --- | --- | --- | --- | --- | --- |
| Cluster 1 vs Cluster 2 | 0.213 | 0.286 | 0.548 | 0.099 | 0.089 |
| Cluster 1 vs Cluster 3 | 0.638 | 0.433 | 0.723 | 0.661 | 0.240 |
| Cluster 1 vs Cluster 4 | 0.482 | 0.003* | 0.317 | 0.772 | 0.921 |
| Cluster 1 vs Cluster 5 | 0.301 | 0.137 | 0.064 | 0.799 | **0.001**** |
| Cluster 1 vs Cluster 6 | 0.133 | 0.051 | 0.282 | **0.001**** | 0.532 |
| Cluster 1 vs Cluster 7 | 1.000 | 0.369 | 0.002* | 0.247 | 0.615 |
| Cluster 1 vs Cluster 8 | 0.610 | **0.001**** | **0.001**** | **0.001**** | **0.001**** |
| Cluster 2 vs Cluster 3 | 0.125 | 0.428 | 0.282 | 0.345 | 0.264 |
| Cluster 2 vs Cluster 4 | 0.800 | 0.364 | 0.089 | 0.261 | 0.536 |
| Cluster 2 vs Cluster 5 | 0.088 | 0.320 | 0.022* | 0.748 | 0.041* |
| Cluster 2 vs Cluster 6 | 0.263 | 0.031* | 0.371 | 0.017* | 0.757 |
| Cluster 2 vs Cluster 7 | 0.351 | 0.723 | **0.001**** | 0.162 | 0.776 |
| Cluster 2 vs Cluster 8 | 0.127 | 0.011* | 0.008* | 0.007* | **0.001**** |
| Cluster 3 vs Cluster 4 | 0.355 | 0.012* | 0.580 | 0.715 | 0.879 |
| Cluster 3 vs Cluster 5 | 0.209 | 0.412 | 0.108 | 0.724 | 0.030* |
| Cluster 3 vs Cluster 6 | 0.083 | 0.252 | 0.235 | **0.001**** | 0.526 |
| Cluster 3 vs Cluster 7 | 0.679 | 0.445 | 0.008* | 0.132 | 0.584 |
| Cluster 3 vs Cluster 8 | 0.921 | **0.001**** | **0.001**** | **0.001**** | **0.001**** |
| Cluster 4 vs Cluster 5 | 0.123 | 0.505 | 0.200 | 0.488 | 0.062 |
| Cluster 4 vs Cluster 6 | 0.226 | **0.001**** | 0.085 | 0.068 | 0.616 |
| Cluster 4 vs Cluster 7 | 0.440 | 0.248 | 0.039* | 0.113 | 0.716 |
| Cluster 4 vs Cluster 8 | 0.344 | 0.038* | 0.221 | 0.067 | 0.022* |
| Cluster 5 vs Cluster 6 | 0.029* | 0.061 | 0.009* | 0.465 | 0.133 |
| Cluster 5 vs Cluster 7 | 0.476 | 0.326 | 0.008* | 0.481 | 0.102 |
| Cluster 5 vs Cluster 8 | 0.218 | 0.986 | 0.417 | 0.980 | 0.275 |
| Cluster 6 vs Cluster 7 | 0.121 | 0.061 | 0.099 | 0.337 | 0.680 |
| Cluster 6 vs Cluster 8 | 0.081 | **0.001**** | 0.053 | **0.001**** | 0.115 |
| Cluster 7 vs Cluster 8 | 0.697 | 0.284 | **0.001**** | 0.547 | 0.073 |

Note: ** p < 0.00178 (significant after Bonferroni correction for 28 comparisons).

* p < 0.05 (significant before correction, but not after Bonferroni). All other p-values ≥ 0.05.

Cluster labels: 1 = Active with short time; 2 = Active with low income; 3 = Active with fear of enclosed places;

4 = Young people with no interest in exercise; 5 = Inactive depressive women; 6 = Active depressive women;

7 = Inactive with money and needs; 8 = Super active.
